# CPMKG: a condition-based knowledge graph for precision medicine

**DOI:** 10.1093/database/baae102

**Published:** 2024-09-27

**Authors:** Jiaxin Yang, Xinhao Zhuang, Zhenqi Li, Gang Xiong, Ping Xu, Yunchao Ling, Guoqing Zhang

**Affiliations:** National Genomics Data Center & Bio-Med Big Data Center, Chinese Academy of Sciences Key Laboratory of Computational Biology, Shanghai Institute of Nutrition and Health, University of Chinese Academy of Sciences, Chinese Academy of Sciences, Shanghai 200031, China; National Genomics Data Center & Bio-Med Big Data Center, Chinese Academy of Sciences Key Laboratory of Computational Biology, Shanghai Institute of Nutrition and Health, University of Chinese Academy of Sciences, Chinese Academy of Sciences, Shanghai 200031, China; Shanghai Information Center for Life Sciences, Shanghai Institute of Nutrition and Health, University of Chinese Academy of Sciences, Chinese Academy of Sciences, Shanghai 200031, China; Shanghai Southgene Technology Co., Ltd., Shanghai 201203, China; Shanghai Information Center for Life Sciences, Shanghai Institute of Nutrition and Health, University of Chinese Academy of Sciences, Chinese Academy of Sciences, Shanghai 200031, China; National Genomics Data Center & Bio-Med Big Data Center, Chinese Academy of Sciences Key Laboratory of Computational Biology, Shanghai Institute of Nutrition and Health, University of Chinese Academy of Sciences, Chinese Academy of Sciences, Shanghai 200031, China; National Genomics Data Center & Bio-Med Big Data Center, Chinese Academy of Sciences Key Laboratory of Computational Biology, Shanghai Institute of Nutrition and Health, University of Chinese Academy of Sciences, Chinese Academy of Sciences, Shanghai 200031, China; Shanghai Sixth People’s Hospital, Shanghai 200233, China

## Abstract

Personalized medicine tailors treatments and dosages based on a patient’s unique characteristics, particularly its genetic profile. Over the decades, stratified research and clinical trials have uncovered crucial drug-related information—such as dosage, effectiveness, and side effects—affecting specific individuals with particular genetic backgrounds. This genetic-specific knowledge, characterized by complex multirelationships and conditions, cannot be adequately represented or stored in conventional knowledge systems. To address these challenges, we developed CPMKG, a condition-based platform that enables comprehensive knowledge representation. Through information extraction and meticulous curation, we compiled 307 614 knowledge entries, encompassing thousands of drugs, diseases, phenotypes (complications/side effects), genes, and genomic variations across four key categories: drug side effects, drug sensitivity, drug mechanisms, and drug indications. CPMKG facilitates drug-centric exploration and enables condition-based multiknowledge inference, accelerating knowledge discovery through three pivotal applications. To enhance user experience, we seamlessly integrated a sophisticated large language model that provides textual interpretations for each subgraph, bridging the gap between structured graphs and language expressions. With its comprehensive knowledge graph and user-centric applications, CPMKG serves as a valuable resource for clinical research, offering drug information tailored to personalized genetic profiles, syndromes, and phenotypes.

**Database URL**: https://www.biosino.org/cpmkg/

## Introduction

The primary challenge in drug therapy is the wide variation in individual responses to medications. This is due to differences in drug metabolism and physiological conditions [1–3]. Precision medicine, unlike the traditional one-size-fits-all approach, tailors treatments to each patient’s unique genetic makeup and health profile [4, 5]. It recognizes the individuality of each person, customizing therapies accordingly. Currently, the most accurate drug information is found on labels and in the Clinical Pharmacogenetics Implementation Consortium (CPIC) guidelines, which explain how genomic data can inform decisions on dosages, metabolism, and potential adverse reactions to certain drugs [6, 7]. However, the FDA has detailed genomic information for only ∼380 drugs on their labels [8]. A significant portion of precision medication data remains buried in basic drug research databases and academic literature [9].

Existing data resources like PharmGKB [10], focusing on pharmacology and pharmacogenomics, CTD [11], with its specialization in toxicological data, and DrugBank [12], offering comprehensive drug information, contain a wealth of drug-related knowledge. However, their usefulness is hindered by a lack of a unified knowledge representation framework and their scattered presence across various platforms. This fragmentation makes it difficult for researchers and clinicians to fully utilize these resources [13]. Other knowledge resources such as the Precision Medicine Knowledgebase (PreMedKB) [14] consolidate ∼500 000 structured precision medicine relationships. But a detailed analysis shows that many of these relationships are oversimplified, labeled merely as “associate” or “effect,” and fail to reflect the nuanced information in the original text. This issue arises from the traditional reliance on “triples” in knowledge graphs as the basic unit of knowledge representation. Traditional triples—comprising a subject, predicate, and object—are limited in conveying complex biomedical information, especially in representing the conditions for the establishment of knowledge [15]. They fail to capture the detailed genomic context required for precision medicine. Therefore, there is an urgent need for methods that can more accurately represent precision medicine knowledge by integrating these genetic conditions into knowledge representation. Furthermore, strategies in integration, mining, and governance should be developed to generate knowledge resources that better meet the requirements of precision medicine. Such resources would resolve ambiguities in current knowledge bases and focus on precise and accurate knowledge representation and dissemination.

To tackle existing challenges in precision medicine knowledge representation, we have developed Condition-based Precision Medicine Knowledge Graph (CPMKG), a comprehensive and advanced knowledge graph based on conditional precision medicine data. Our achievements include the following:

- We introduced the “Hyper-Triple,” a novel framework that redefines the core data units in knowledge graphs. This framework overcomes the limitations of traditional triples by incorporating specific conditions (genetic backgrounds), ensuring that certain relationships are valid only under specific circumstances. This approach enhances the accuracy and depth of our knowledge representation, distinguishing CPMKG from traditional knowledge graphs.
- We developed a “knowledge pattern” approach for organizing data. This method summarizes events involving multiple entities into a model, serving as an abstraction of conditional domain knowledge derived from the literature or expert input. These patterns are represented using the “Hyper-Triple” framework, guiding the collection of domain-specific knowledge.
- CPMKG defines four key knowledge patterns for precision medicine research and application. It integrates 307 614 pieces of knowledge, addressing the needs of personalized medicine and drug discovery. The graph presents explicit relationships and constraints, enhancing the precision of therapies.
- The knowledge graph offers a drug-centric exploration landscape, merging insights from molecular and clinical research into a comprehensive reasoning map. This facilitates the discovery of valuable evidence, supports medication synergy, and incorporates pharmacogenomics for holistic drug recommendations.
- CPMKG employs a large language model (LLM) to improve understanding of the knowledge graph. It provides clear explanations for subgraphs generated through system reasoning, balancing structured information with language expression for better user comprehension.

## Materials and methods

### Data collection

To delve into precision medicine knowledge, CPMKG has aggregated and restructured 15 727 studies from nine key drug-related databases: PharmGKB [10], SIDER [16], CIViC [17], DrugBank [12], TTD [18], CTD [11], DCDB [19], DoCM [20], and PharmacotherapyDB (https://github.com/dhimmel/indications). We meticulously extracted and mined information on relationships between various entities, such as drug side effects, sensitivities, molecular mechanisms, and treatments.

### Knowledge pattern and conditional knowledge representation

#### CPMKG knowledge pattern

Biomedical literature serves as a vital repository of knowledge, where authors craft sentences to define and delineate key concepts. However, not every sentence contains critical information. Effective distillation of pertinent knowledge enhances the precision of text mining tools, bolstering their ability to expand knowledge bases and clarify the application scope of knowledge graphs. Our research primarily examines pharmacogenomics and drug research papers. In clinical drug studies, the focus often lies on treatment and side effects, whereas drug discovery research prioritizes understanding drug sensitivity and mechanisms. Despite varying linguistic expressions, these knowledge sources share semantic and entity-level commonalities. We harness these similarities through knowledge patterns to capture essential information accurately.

**Figure 1.**
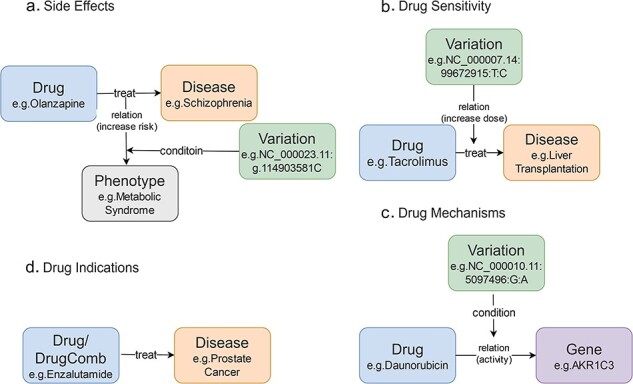
Knowledge patterns in CPMKG. (a) Side effects refer to side effects or complications that occur during the use of medication. Example: association between olanzapine and metabolic syndrome risk in schizophrenia patients. (b) Drug sensitivity refers to an individual’s propensity to exhibit a heightened or exaggerated response to medication compared to the average population. Example: influence of the CC genotype on tacrolimus requirement in liver transplant patients. (c) Drug mechanism refers to the relationship between an individual’s genome and their response to medications. Example: reduction of AKR1C3 enzyme activity during daunorubicin treatment in patients with the A allele variant. (d) Drug indication refers to the formal recommendation for medication use in treating specific diseases or pathological conditions. Example: utilization of enzalutamide in prostate cancer treatment.

In CPMKG, knowledge is organized into four distinct patterns: Pattern 1 links drug side effects to disease treatment, highlighting genetic variations that signal increased risk (e.g. the C allele of olanzapine and metabolic syndrome in schizophrenia, Fig. 1a) [21–23]. Pattern 2 focuses on drug sensitivity, showing how genetic variations impact treatment outcomes (e.g. reduced tacrolimus need in liver transplant patients with the CC genotype, Fig. 1b) [24–26]. Pattern 3 elucidates drug mechanisms by connecting drug usage to changes in gene expression influenced by genetic variations (e.g. reduced AKR1C3 enzyme activity with the A allele during daunorubicin treatment, Fig. 1c) [27]. Pattern 4 outlines drug indications, providing therapeutic insights for specific drugs or combination therapies (e.g. enzalutamide for prostate cancer, Fig. 1d) [28]. These knowledge patterns often use genetic variations as conditions, highlighting the need for a more nuanced representation.

#### Conditional knowledge representation framework and storage

The knowledge patterns we designed, including node-to-edge (e.g. conditional) and edge-to-edge (e.g. causal) connections, cannot be intrinsically represented by conventional knowledge graphs, which only support node-to-node triples. To address this limitation, we extend to a “hypergraph” that accommodates complex information and allows for these non-node-to-node connections. In our knowledge graph, tuples can denote relationships between both entities and relations. For a tuple (S, R, and O), where S, R, and O stand for subject, relation, and object, respectively, R acts as a predicate, while S and O can be entities or other tuples. This structure is defined as follows:


$$G = {\ }\langle V,E\rangle \\[-6pt]$$

$$E = {E_{{\mathrm{vv}}}},{E_{{\mathrm{ev}}}},{E_{{\mathrm{ee}}}}$$

Here, ${E_{{\mathrm{vv}}}}$ represents edges that connect a vertex or an entity to another. ${E_{{\mathrm{ev}}}}$ denotes edges that link a vertex to an edge or vice versa. Finally, ${E_{{\mathrm{ee}}}}$ signifies edges that connect two edges. In a semantic context, ${E_{ev}}$ is often utilized to denote a constraint condition for a tuple, while ${E_{ee}}$ usually describes how one tuple (or event) leads to another. This framework allows for a more nuanced representation of complex relationships and conditions within the knowledge graph (Supplementary Fig. S2).

Our graph structure incorporates causes, conditions, and other crucial information, enabling detailed exploration of relationships. This intricate hypergraph comprises three fundamental structures: node-to-node, node-to-relation, and relation-to-relation connections, as shown in Supplementary Fig. S1a–c. Our knowledge representation framework also includes concept composition, representing combined medication as a collective “ALL” union of various drugs. We introduce “gate” nodes, inspired by logic gates, to amalgamate concepts and relations into new entities. We use two primary gates: the “AND gate,” integrating all members, and the “OR gate,” combining some members (see Supplementary Fig. S1d). Multiple nodes or edges directly connected without a gate node are considered independent. Additionally, our framework allows for the expression of negation and likelihood in all relations, making it highly expressive and adaptable for various scenarios.

Due to our updated knowledge representation, the classic knowledge graph storage method cannot accommodate our framework. To address this, we can adapt the data structure to better fit the storage capabilities of contemporary graph databases. Our approach involves integrating a helper node within a relationship, serving as a meaningful predicate. Specifically, we augment relationship predicates to function similarly to entities. We introduce a special relationship node that represents the original relationship predicate. This node uses “from” and “to” edges to indicate the direction of the relationship between the subject and object nodes. As a result, a triple’s relationship is routed through this node, facilitating connections to entities or other triples. Furthermore, we can insert an auxiliary node within relationships to convey complex relationships more effectively, as demonstrated in Supplementary Fig. S2D. Additionally, this method enhances the flexibility and scalability of our knowledge graph, allowing for more intricate data representations and improving query performance.

### Conditional knowledge mining

To ensure better alignment with our knowledge patterns, we refined our approach using several methods for knowledge mining and data integration.

We employed automated entity and relationship extraction techniques, utilizing regular expressions to parse unstructured text from databases such as PharmGKB [10], DrugBank [12], and CTD [11]. This approach allowed us to identify and extract entities and their relationships, converting raw text into structured data for our knowledge patterns. For a comprehensive description, refer to the Supplementary Methods for Processing Each Database.

From PharmGKB [10], we extracted 13 055 entries, and from CTD [11], we extracted 143 280 entries, which were then standardized and integrated.

To create a unified dataset for databases like SIDER [16], where data were scattered across multiple files, we merged and standardized various data tables. This process ensured consistency and completeness, resulting in a consolidated dataset of 89 491 entries.

For databases like TTD [18], which provided data in HTML format, we extracted relevant information by parsing the HTML content and reorganized it into a standardized dataset format, processing a total of 1002 entries.

In databases like PharmacotherapyDB (https://github.com/dhimmel/indications), where files contained duplicates, we performed deduplication by comparing data points and removing redundant entries. This process ensured each entry was unique and accurate, resulting in 11 751 unique entries post-deduplication.

For databases like DCDB [19], which listed multiple drugs in a single table, we isolated each drug entry and combined relevant data points to form a comprehensive dataset, processing 496 entries.

For databases like CIViC [17] and DoCM [20], which lacked structured relationships or complete data, we employed manual curation. Experts identified and added missing entities and relationships to ensure completeness and accuracy. This resulted in the curation of 1998 entries from CIViC [17] and 89 entries from DoCM [20], filling gaps and ensuring accurate representation.

### Graph interpretation by LLM

To improve the user experience of CPMKG, we have incorporated the ChatGPT API, specifically the gpt-4o, into our web application. The integration of the AI-generated content model equips our web application with advanced natural language processing capabilities, significantly enhancing its interactivity and intelligence. We have designed prompts for four distinct application scenarios (Supplementary Table S3). The graphic descriptions generated in response to these prompts are tailored to user needs and supported by evidence from our knowledge graph. In this setup, the LLM refines and consolidates our knowledge, producing content that is both user-friendly and accurate. Importantly, we ensure that the model strictly adheres to the information in the knowledge graph, preventing the introduction of unsupported information and avoiding the risk of model hallucination.

### Entity disambiguation

Entity disambiguation is a critical process for resolving ambiguities among entities sharing the same name. In CPMKG, this technique is applied to standardize five distinct types of entities: drugs, diseases, phenotypes, genetic variations, and genes. Each of these entities is associated with its own ontology, encompassing controlled vocabularies of standard names, synonyms, and IDs (the ontologies used for these entities are detailed in Supplementary Table S2).

For entities where a direct correlation exists between the source database entity ID and the target ontology ID, we employ ID mapping for straightforward standardization. For entities lacking this direct link, name mapping is utilized. Using controlled vocabulary *M* = (*m*1, *m*2, …, *mn*), we map the original structured names *N* of these entities against *M* to identify the most accurate terms. When this mapping results in a unique ID, it is adopted as the entity’s external ID. In cases where the mapping leads to multiple “best matches,” manual correction is undertaken.


$${{{\Gamma }}_{{\mathrm{best}}}} = {\mathrm{argma}}{{\mathrm{x}}_{{\Gamma }}}\mathop {\mathop \sum}\limits^n_{i = 0} \varphi ({m_i},N)$$

## Results

### Statistics on entities and knowledge in CPMKG

CPMKG aims to advance precision medicine and drug discovery in clinical research. Utilizing a unified knowledge representation framework, CPMKG consolidates comprehensive pharmaceutical knowledge through processes like knowledge acquisition, element mining, and restructuring (Supplementary Fig. S3). This process has yielded 307 614 pieces of detailed drug knowledge, including 139 824 entries on side effects, 9819 on drug sensitivity, 144 269 on drug mechanisms, and 13 702 on drug indications. This comprehensive data encompasses 2150 drugs, 1689 diseases, 1719 phenotypes, 5029 genetic variations, and 20 111 genes (Table 1). For a more detailed statistical breakdown, including filtering and merging across various databases, refer to Supplementary Table S1.

**Table 1.** Statistics on entities and knowledge in CPMKG

Knowledge pattern	Knowledge source	Knowledge	Drug	Disease	Phenotype	Variant	Gene
Side effects	SIDER, PharmGKB, and DrugBank	139 824	1712	544	1719	1447	597
Drug sensitivity	CIViC, TTD, DrugBank, DoCM, and PharmGKB	9819	496	336	–	3478	880
Drug mechanisms	CIViC, CTD, and PharmGKB	144 269	1039	–	–	682	19 984
Drug indications	PharmacotherapyDB, DCDB, and SIDER	13 702	1394	1544	–	–	–
Total	–	307 614	2150	1689	1719	5029	20 111

En-dashes (–) : data is not available for this knowledge pattern.

In terms of entity disambiguation, the process resulted in the standardization of 30 698 entities, with 918 entities not aligned with external database mappings. Notably, there are 618 entities in CPMKG that can be classified as both diseases and phenotypes. These entities are represented in different knowledge patterns: diseases are associated with treatments, while phenotypes are linked to side effects. Users can choose the appropriate classification based on their specific use case.

Unlike traditional methods that integrate databases from diverse sources, CPMKG focuses on aligning data sources to predefined knowledge patterns. This methodology involves literature mining and manual curation based on original evidence within these patterns. This strategy not only gathers essential knowledge elements, such as drug interactions and genomic variations, but also enhances existing data by introducing new knowledge patterns.

### Conditional knowledge-based schema of CPMKG

**Figure 2.**
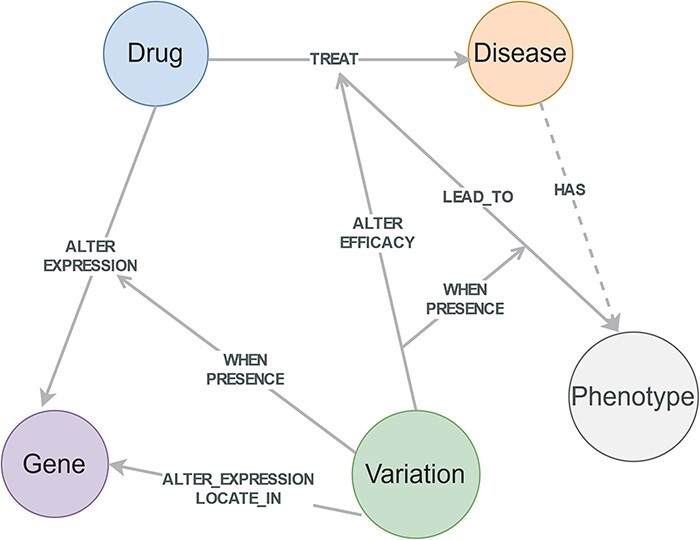
Conditional knowledge-based schema of CPMKG. This schema includes foundational elements such as drugs, diseases, phenotypes, genes, and variations. “Drugs” cover pharmacological substances, “diseases” encompass pathological conditions, “variations” refer to differences in the human genome, “phenotypes” include side effects or complications, and “genes” pertain to human genes. This schema illustrates the integration of these entities and their detailed relationships, highlighting the four conditional knowledge patterns in precision medicine.

The conditional knowledge-based schema of CPMKG is constructed from four primary knowledge patterns: drug side effects, drug sensitivity, drug mechanisms, and drug indication. These patterns form the foundational elements of the schema, including entities such as drugs, diseases, phenotypes, genes, and variations. The relationships among these entities establish the schema’s structure (Fig. 2). Differing from traditional knowledge graphs, CPMKG incorporates critical yet long-missing causal and conditional associations, embodied in relationships between entities and triples, as well as among triples themselves. This allows for a nuanced representation of precision medicine knowledge, highlighting differences in individual genetic backgrounds and population characteristics.

### Drug-centered conditional knowledge exploration

**Figure 3.**
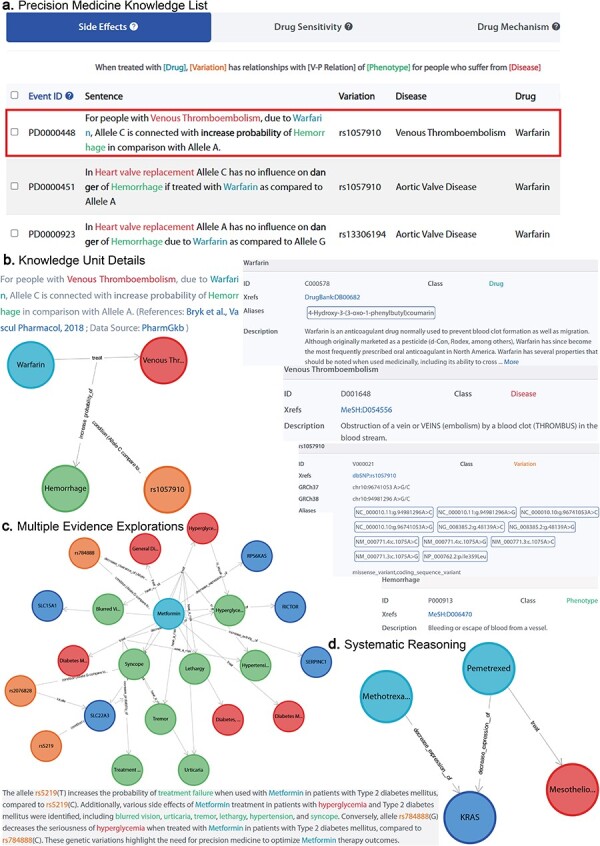
Knowledge exploration in CPMKG. (a) Precision medicine knowledge list. A list centered on “warfarin,” comprising four distinct patterns. (b) Knowledge unit details. Illustrated by “warfarin treatment side effects,” it includes graphical representation, established conditions, evidence sources, and entity details. (c) Multiple evidence explorations. Subgraph exploration centered on metformin, along with knowledge description. (d) Systematic reasoning. Illustration of pemetrexed’s efficacy in MPM treatment and its correlation with reduced *KRAS* expression, suggesting a shared mechanism with methotrexate, supporting methotrexate’s potential effectiveness in MPM treatment.

CPMKG empowers researchers to delve into drug-centered research in precision medicine. It offers users access to knowledge across four categories, including medication recommendations and pharmacogenomics, anchored in core elements like drugs and genetic variations. For example, Fig. 3a displays a knowledge list centered on “warfarin.” It provides switchable lists covering four types of precision medicine knowledge, enabling users to deeply understand and compare warfarin’s effects across different genetic backgrounds and explore personalized drug recommendations via entity-condition-relationship pairs.

Furthermore, CPMKG presents each knowledge unit graphically, allowing users to visualize the type of knowledge, the conditions under which it was established, and its evidence sources within the knowledge graph. This is accompanied by detailed knowledge descriptions and annotations for each entity. For instance, Fig. 3b demonstrates that under the genetic background *NC_000010.11:g.94981296A>C*, warfarin treatment for venous thromboembolism heightens bleeding risk [29]. This graphical representation provides an intuitive understanding of the knowledge, while the descriptions and entity annotations offer an in-depth comprehension of the graph.

### Knowledge inference with multiple evidence

Each knowledge unit, representing a specific knowledge pattern, can effectively communicate the author’s intended information. However, the scope of knowledge conveyed by a single piece of literature remains confined. CPMKG, as a knowledge graph, empowers researchers to integrate multiple knowledge instances into subgraphs, each drawing on numerous evidence sources. This allows for systematic reasoning about pertinent knowledge connections within these subgraphs.

Take, for example, the frequent occurrence of *KRAS mutations* (*KRAS* is a proto-oncogene that encodes a GTPase) in cancer, a factor in >20% of human cancers. These mutations are also present in patients with malignant pleural mesothelioma (MPM). As shown in Fig. 3d, pemetrexed effectively treats MPM and reduces KRAS protein expression, a mechanism shared with methotrexate [30]. This shared mechanism led us to hypothesize methotrexate’s effectiveness in treating MPM, a hypothesis supported by the literature [31].

To make these subgraphs more accessible, we have innovatively combined LLMs with precise, specialized medical knowledge from our database, including reference articles, to provide clear descriptions for each subgraph. We offer four distinct scenarios for varied graph descriptions, skillfully connecting structured graphs with narrative language. For instance, Fig. 3c displays a subgraph centered on “metformin,” illustrating gene variations and their impact on diabetic patients’ responses and side effects. The *rs784888(G)* allele correlates with a better response to metformin, reducing hyperglycemia severity compared to *rs784888(C)* [32], while the *rs5219(T)* allele is linked to an increased likelihood of treatment failure compared to *rs5219(C)* [33]. Common side effects for hyperglycemia patients taking metformin include blurred vision, urticaria, pruritus, skin rash, tremor, lethargy, hypertension, and syncope.

### Advanced application of CPMKG (case study)

CPMKG enhances the drug usage experience with three user-centric applications: personalized drug suggestion, which offers tailored medical advice; pharmacogenomics application, accelerating drug mechanism research and discovering new applications for existing drugs; and medication synergy assistant, aiding in the selection of effective drugs or drug combinations.

#### Case study 1: personalized drug suggestion

**Figure 4.**
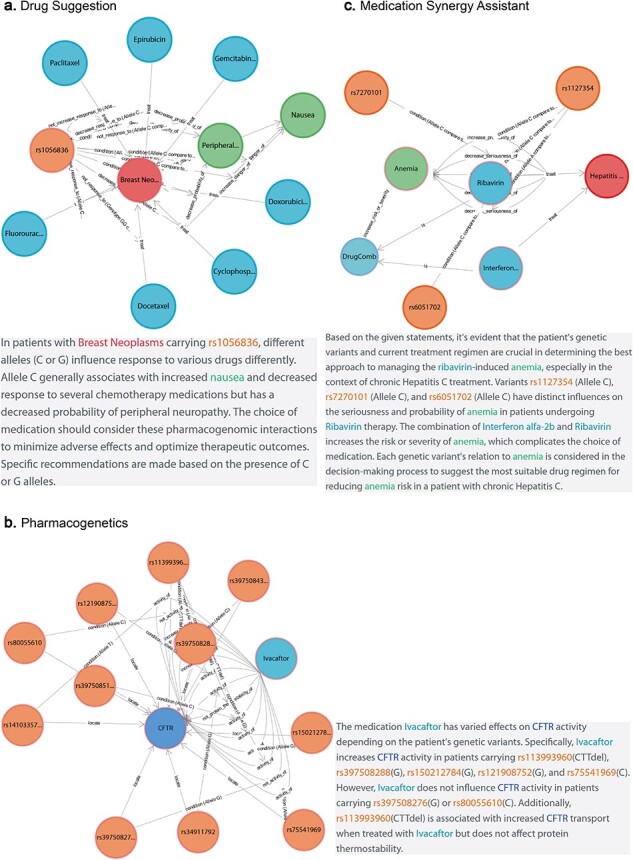
Advanced application of CPMKG. (a) Personalized drug suggestion offers tailored medical advice based on diagnostic outcomes and genetic backgrounds. Example: crucial factors for prescribing medication to breast cancer patients with the *NC_000002.12:g.38071060G>A,C* variant. (b) Pharmacogenomics focuses on understanding drug mechanisms for personalized medicine and novel drug discovery. Example: ivacaftor’s effects on various *CFTR* alleles and genotypes in CF. (c) Medication synergy assistant optimizes treatment outcomes and patient safety, particularly with multiple drugs. Example: effects of interferon α-2b and ribavirin in treating chronic hepatitis C.

Personalized drug suggestion aids clinical research by enabling individualized medical advice based on patients’ diagnostic outcomes and genetic backgrounds. Consider Fig. 4a, which delineates crucial factors for prescribing medication to breast cancer patients carrying the *NC_000002.12:g.38071060G>A,C* genetic variant. For the GG genotype, docetaxel may be ineffective [34]. In contrast, the CC genotype can increase nausea risk with doxorubicin [35] and potentially lead to diminished efficacy with epirubicin [36]. However, this variant does not impact the effectiveness of gemcitabine and paclitaxel [37]. Importantly, cyclophosphamide has the potential to reduce peripheral neuropathy risk in patients with the C allele [38]. CPMKG provides valuable genotype-specific references to support prescription choices in clinical research.

#### Case study 2: pharmacogenomics application

Pharmacogenomics plays a crucial role in understanding drug mechanisms for personalized medicine and novel drug discovery, particularly in diseases like cystic fibrosis (CF). CF, caused by mutations in the CF transmembrane conductance regulator (*CFTR*) gene [39], can be treated with ivacaftor, a CFTR potentiator that enhances CFTR protein function [40]. Figure 4b demonstrates ivacaftor’s effects on various *CFTR* alleles and genotypes, highlighting 11 genomic variations that significantly influence the drug’s pharmacological response in the human body. For instance, ivacaftor treatment alters *CFTR* activity in the *NC_000007.14:g.117603654T>A,C* and *NC_000007.14:g.117611620A>C* variants. Additionally, the *NC_000007.14:g.117559592_117559594del* variant is linked with increased CFTR transport [41] but does not affect the protein’s thermal stability [42]. Such insights are invaluable for developing targeted treatments for patients with CFTR-related conditions.

#### Case study 3: medication synergy assistant

In drug indication, both efficacy and side effects are of paramount importance. Medication synergy assistants can optimize treatment outcomes and bolster patient safety, particularly when multiple drugs are used, either in combination or individually. For example, Fig. 4c demonstrates the effects of interferon α-2b and ribavirin in treating chronic hepatitis C [43]. Both drugs, whether used separately or together, significantly increase the risk and severity of anemia. However, patients with the *NC_000020.11:g.3271278A>C* and *NC_000020.11:g.3213247A>C* genetic variants experience less severe anemia after ribavirin treatment [44, 45]. Consequently, ribavirin therapy is recommended for patients with these specific genetic profiles.

## Discussion

Despite the advancements achieved with CPMKG, some detailed aspects still require deeper exploration. Precision medicine demands a thorough understanding of both entities and their attributes, such as gene variant genotypes and clinical indices. Transitioning from traditional triples to hyper-triples presents challenges in making accurate inferences due to the detailed and specific conditions involved. However, genetic information in drug databases is limited, and even the original literature often lacks necessary genomic details. As this area is under-researched, we aim to refine this in future studies and encourage broader contributions. This shift also underscores the need for further research into advanced knowledge reasoning methods. Our forward-looking approach leverages the sophisticated understanding capabilities of LLMs to decode complex semantics and hyper-triples. Additionally, we aim to utilize natural language interpretation based on intricate knowledge reasoning, driving the advancement of application-focused knowledge graphs.

## Conclusion

CPMKG revolutionizes traditional drug knowledge by incorporating refined elements like specific conditions, making it ideal for precision medicine. Our knowledge graph offers personalized medication recommendations based on patients’ genetic profiles, serving as a reference for clinical practice. It also supports researchers by facilitating drug metabolism studies and targeted drug discovery. Unique in its approach, CPMKG employs the “hyper-triple” concept in knowledge representation, capturing the complex nuances of precision medicine with remarkable accuracy. It merges and rationalizes various precision medicine knowledge pieces through innovative knowledge graph construction methods. This process not only uncovers information overlooked in current research but also enhances the understanding and application of these knowledge graphs in clinical research. Furthermore, the hypergraph structure can be seamlessly integrated into any graph database, accommodating existing database technologies while ensuring minimal information loss compared to the original research publications. This effectively preserves the depth and complexity of the relationships, providing a robust and comprehensive foundation for future clinical-related research.

To make our knowledge graph more user-friendly, we have integrated LLM for graph interpretation. This integration not only advances our construction methods but also enriches the fusion of structured graphs with textual data. It broadens the spectrum of user engagement with knowledge graphs, paving the way for new perspectives in their representation, storage, and interpretation.

## Supplementary Material

baae102_Supp

## Data Availability

CPMKG is publicly accessible through https://www.biosino.org/cpmkg/. All data and resources hosted on the platform are freely accessible.
