# Supplementary material for: CPMKG: a condition-based knowledge graph for precision medicine
Source: Database (Oxford). 2024 Sep 27;2024:baae102. doi: 10.1093/database/baae102 (PMC11429523; doi:10.1093/database/baae102)
Supplement: baae102_Supp [file baae102_supp.zip › Supplementary_0917.docx]

# Supplementary Material


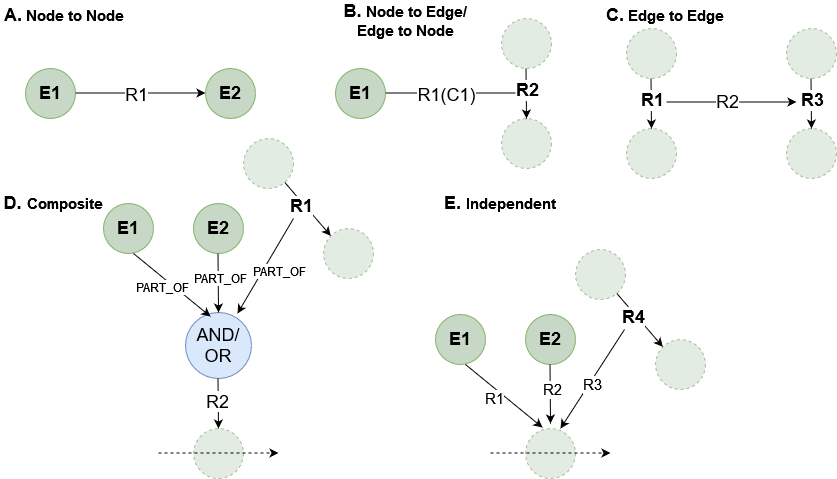


**Figure S1.** Hypergraph structures in biomedical knowledge representation. (A-C) Node-node, node-relation, relation-relation structures to extend conventional knowledge graph to a knowledge hypergraph. (D) Composite of various hypergraph components, a “all” or “one” composite can be expressed like so. (E) Independent hypergraph component that related with a node or relation.


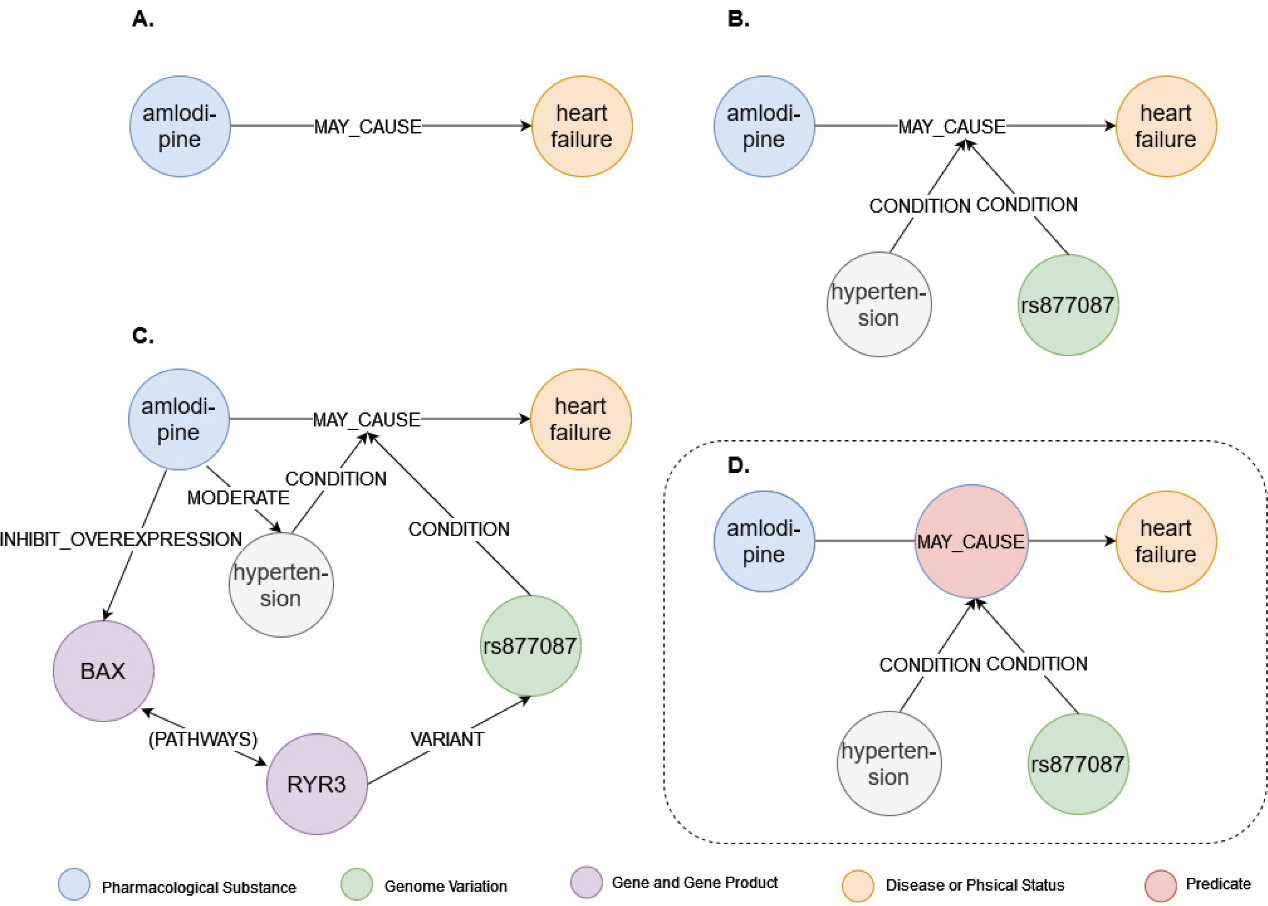


**Figure S2.** Enhanced representation of conditional relationships in biomedical knowledge. (A) Simple triple representation of an event (amlodipine, MAY_CAUSE, heart failure). (B) Hyper representation of the same event contains more information including the valuable conditional constraints. (C) Representing conditions as individual nodes allows easy extension of linking related entities and events. (D) Data structure used to store proposed hypergraph model in conventional graph databases.


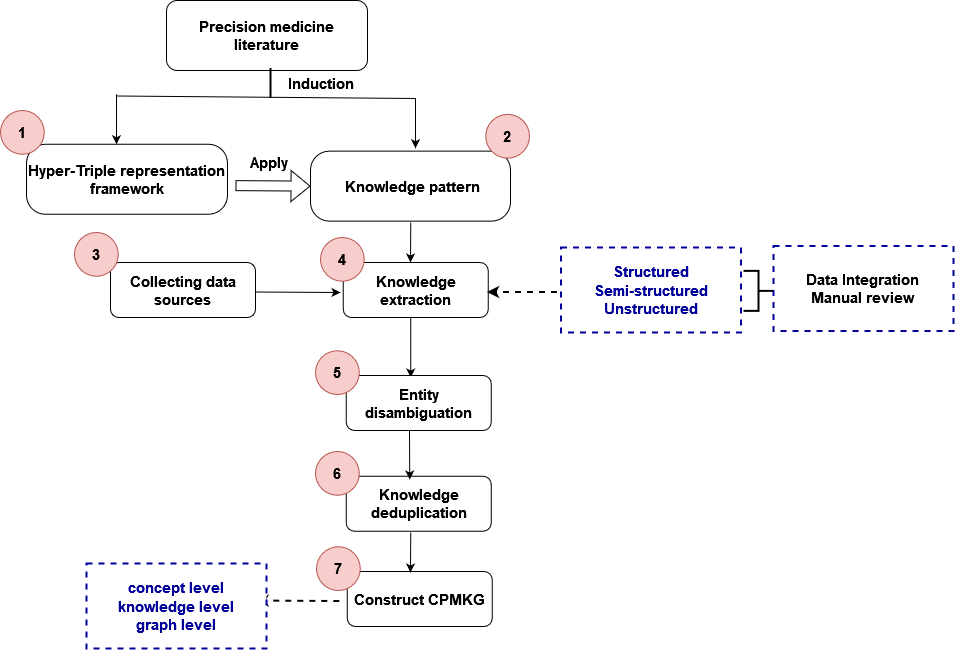


**Figure S3.** Workflow of CPMKG Construction.

**Table S1.** Detailed statistical breakdown for each database.

| **DataBase** | **Original Total** | **Filtered** | **Retained** | **Unique** | **Merged** | **Condition** | **Side Effects** | **Drug Sensitivity** | **Drug Mechanism** | **Drug Indication** |
| --- | --- | --- | --- | --- | --- | --- | --- | --- | --- | --- |
| PharmGkb | 18,846 | 5,123 | 13,723 | 13,055 | 668 | 13,055 | 5,475 | 6,640 | 940 | \ |
| SIDER | 147,227 | 57,614 | 89,613 | 89,491 | 122 | 0 | 88,036 | \ | \ | 1,455 |
| CIViC | 2,586 | 557 | 2,029 | 1,998 | 31 | 1,998 | \ | 1,949 | 49 | \ |
| DrugBank | 212,681 | 144,104 | 68,577 | 46,452 | 22,125 | 139 | 46,313 | 139 | \ | \ |
| TTD | 3,528 | 2,367 | 1,161 | 1,002 | 159 | 1,002 | \ | 1,002 | \ | \ |
| CTD | 1,214,204 | 1,070,854 | 143,350 | 143,280 | 70 | 0 | \ | \ | 143,280 | \ |
| PharmacotherapyDB | 16,204 | 3,882 | 12,322 | 11,751 | 571 | 0 | \ | \ | \ | 11,751 |
| DrugCombDB | 1,560 | 1,060 | 500 | 496 | 4 | 0 | \ | \ | \ | 496 |
| DoCM | 183 | 51 | 132 | 89 | 43 | 89 | \ | 89 | \ | \ |
| Total | 1,617,019 | 1,285,612 | 331,407 | 307,614 | 23,793 | 16,283 | 139,824 | 9,819 | 144,269 | 13,702 |

**Table S2.** Entity standardization databases of CPMKG.

| **Entity type** | **Standardization database(s)** |
| --- | --- |
| Drug | DrugBank(https://go.drugbank.com/), PubChem(https://pubchem.ncbi.nlm.nih.gov/) |
| Disease | Disease Ontology(https://disease-ontology.org/), MeSH(https://www.ncbi.nlm.nih.gov/mesh/) |
| Phenotype | Human Phenotype Ontology(https://hpo.jax.org/app/), MeSH(https://www.ncbi.nlm.nih.gov/mesh/) |
| Variation | dbSNP(https://www.ncbi.nlm.nih.gov/snp/ |
| Gene | NCBI Gene(https://www.ncbi.nlm.nih.gov/gene/) |

| **Table S3.** Application scenarios and prompts for ChatGPT integration. | |
| --- | --- |
| **Application Scenarios** | **Prompts** |
| Personalized Drug Suggestion | You are an experienced doctor who is excellent at precision medicine researches. I have a patient carrying the <variant1>,<variant2> and <variant_...> having <disease1>,<disease2> and <disease_...>. I'm considering some drugs for this patient. Below are some statements from various studies to help us making the best drug suggestion for this patient, which one should we choose, give different suggestions based on the assumption of the allele or genotype this patient carries? First, write a truthful and detailed tl;dr (~500 words) of the relations and knowledge of the provided statements and DO discuss different cases. Then analyze them step by step and finally summarize the reasoning stages into a (~300 words) finalized medication prescription suggestion with a simple reason. |
| Medication Synergy Assistant | You are an experienced doctor who is excellent at precision medicine researches. I have a patient experiencing <symptom_1>, <symptom_2> and <symptom_...>, and taking the <drug_1>, <drug_2> and <drug_...>. Given that some drug combination is beneficial while others may be lethal. I'm considering some drugs for this patient. Below are some statements from various studies to help us making the best drug suggestion for this patient, which one should we choose? First, write a truthful and detailed tl;dr (~500 words) of the relations and knowledge of the provided statements and DO discuss different cases. Then analyze them step by step and finally summarize the reasoning stages into a (~300 words) finalized medication prescription suggestion with a simple reason. |
| Pharmacogenomics | You are an experienced doctor who is excellent at precision medicine researches. Below are some statements from various studies, I need you to help me understand why the medication may have different effects on patients with or without <variant>, considering the allele or genotype this patient carries. First, write a truthful and detailed tl;dr (~500 words) of the relations and knowledge of the provided statements and DO discuss different cases. Then analyze them step by step and finally summarize the reasoning stages into a (~300 words) finalized medication prescription suggestion with a simple reason. |
| Default | You are an experienced doctor who is excellent at precision medicine researches. Here are some statements from various studies. Please write a truthful and detailed tl;dr (~500 words) of the relations and knowledge of the provided statements and DO discuss different cases. |

**Supplementary Methods for Processing Each Database**

**SIDER - Data Extraction and Integration**

The SIDER database offers detailed drug-phenotype information and drug labels in the meddra_all_label_se file, from which we extracted drug-disease information. We used XPath queries to navigate the HTML structure of drug labels, specifically targeting sections with keywords like "Therapeutic indication" and "INDICATIONS." Disease names were extracted from paragraphs related to treatment.

1. *# XPath extraction for disease-related information*
2. diseases=html.xpath('body//h2[contains(text(),"Therapeutic indication") \
3. or contains(text(),"INDICATIONS") or \
4. contains(text(),"Indications")]/ \
5. ancestor::div[@class="section_ind"]// \
6. p[contains(text(),"Treat") or contains(text(),"treat")]/ \
7. span[1][contains(@class,"h se")]/text()')

This extracted disease information was then integrated into the original drug-phenotype dataset, expanding it into a comprehensive drug-disease-phenotype knowledge base. This integration enriched the dataset, providing a deeper understanding of the relationships between drugs, diseases, and phenotypes. Entities were matched against a standardized knowledge base, and any entries that could not be confidently linked to a standard reference were excluded. After disambiguation, the data was grouped and merged by drug, disease, and phenotype, ensuring each combination was uniquely represented.

**DrugBank - Data Extraction and Integration**

The DrugBank database, available for download in XML format upon obtaining the necessary license, requires parsing to extract meaningful research data.

To efficiently process the XML files, we employed regular expressions (regex patterns) tailored to capture key elements such as drug IDs, names, interactions, and descriptions of pharmacogenomic effects or drug interactions. Additional regex patterns were applied to the description fields to identify and structure specific relationships between drugs and side effects. This method allowed us to extract detailed information on side effects and their associations with drugs. The following sections detail the regex patterns used and the specific information they extracted (Table S4).

**Table S4.** Regular expression patterns for DrugBank data extraction.

| **Information** | **Regex Pattern** | **Example** |
| --- | --- | --- |
| drugbank id | <drug type.*?<drugbank-id primary="true">(.*?)</drugbank-id> | <drugbank-id primary="true">**DB00004**</drugbank-id> |
| drugbank name | <drug type.*?<drugbank-id primary="true">.*?<\/drugbank-id>.*?<name>(.*?)<\/name> | <name>**Denileukin diftitox**</name> |
| snp effect | <effect>.*?<description>(.*?)<\/description>.*?<\/effect> | <description>**Patients with this genotype in IFNL3 have a reduced likelihood of achieving sustained virologic response to peginterferon alfa-2a therapy.**</description> |
| variation | <rs-id>(.*?)<\/rs-id> | <rs-id>**rs396991**</rs-id> |
| drug interaction description | <drug-interaction>.*?<description>(.*?)<\/description>.*?<\/drug-interaction> | <description>**The risk or severity of hypotension and orthostatic hypotension can be increased when Valsartan is combined with Levodopa.**</description> |
| relation | cause\s(a\|an\|the)\s(.*?)\sin\sthe\s(.*?)\sof\s' | Cyclandelate can cause a **decrease** in **the absorption of** Magnesium resulting in a reduced serum concentration and potentially a decrease in efficacy. |
| relation | may\s(.*?)\sof.* | Apixaban may **increase the anticoagulant** **activities** of Lepirudin. |
| relation | \sand.*?(a\|an\|the)\s(.*?)\s(.*?)\. | and potentially a reduction in efficacy |
| relation | The\s(.*?)\sof\s.*?\scan\sbe\s(.*?)d\swhen | The **risk or severity** of bleeding and hemorrhage can be **increased** when Dasatinib is combined with Lepirudin. |
| phenotype | result\sin\s(?:a\|an\|the)\s(\w+)\s(.*)\. | Indomethacin may decrease the excretion rate of Leuprolide which could result in a **higher serum level**. |

**PharmGKB - Data Extraction and Integration**

PharmGKB data files contain sentences documenting relationships between various biomedical entities. To transform this unstructured text into structured knowledge, we applied regular expressions to extract relevant entities and relationships, converting raw text into a format suitable for analysis and research.

The following table details the regular expression patterns used to identify and extract specific entities and relationships from PharmGKB sentences. Each pattern targets different types of information, including drugs, diseases, variants, phenotypes, genes, and their associated relationships.

These regular expressions were carefully crafted to match specific patterns within the sentences, enabling the precise extraction of relevant data for each entity type, such as drugs, diseases, and variants (Table S5).

**Table S5.** Regular expression patterns for PharmGKB data extraction.

| **Information** | **Regex Pattern** | | **Example** |
| --- | --- | --- | --- |
| Drug | (treated with\|exposed to\|due to) (.+?)( in \| as \|\.) | | Allele T is associated with decreased activity of CES1 when treated with **trandolapril** as compared to allele C |
| Disease | in ([\w ]+) with (.+?)( as \|\.) | Allele (CCCACCCGA)10 is not associated with increased risk of Nausea when treated with fluvoxamine in people with **Depressive Disorder, Major** as compared to allele (CCCACCCGA)12. | |
| Variation | ^(.+?) (is\|are) .*?as compared to (.+?)\. | **Allele (CCCACCCGA)10** is not associated with increased risk of Nausea when treated with fluvoxamine in people with Depressive Disorder, Major as **compared to allele (CCCACCCGA)12**. | |
| Phenotype | associated with .*? (\b[A-Za-z]+\b)(?: in \| as \| when \| due to \|\.) | Allele (CCCACCCGA)10 is not associated with increased risk of **Nausea** when treated with fluvoxamine in people with Depressive Disorder, Major as compared to allele (CCCACCCGA)12. | |
| Gene | associated with .*?(\b[A-Z0-9]{2,}\b) | Genotype 12 is associated with increased expression of **SLC6A4**. | |
| Relation | associated with (.+?)( of \| in \| as \| when \| due to \|\.) | Allele (CCCACCCGA)10 is associated with **increased risk of** Nausea when treated with fluvoxamine in people with Depressive Disorder, Major as compared to allele (CCCACCCGA)12. | |

**CTD - Data Extraction and Integration**

The Comparative Toxicogenomics Database (CTD) provides tables for drugs and genes, along with sentences that document the relationships between these entities. To extract drug-gene relationships, we applied regular expressions to identify and capture relevant entities and their interactions within these sentences (Table S6).

Since many CTD entries refer to chemical entities, we performed disambiguation using DrugBank for drugs and HGNC for genes. This involved mapping CTD drug entries to DrugBank for precise drug identification and aligning CTD gene entries with HGNC to confirm gene identities.

After disambiguation, we removed entries that did not correspond to recognized drugs, ensuring that our dataset focused solely on relevant drug-related information. We then integrated the data by drug, gene, and relationship, forming a comprehensive drug-gene-relationship knowledge base. To maintain accuracy, we cross-referenced the extracted data with DrugBank and HGNC, verifying that all relationships and entities were correctly identified and represented.

**Table S6.** Regular expression patterns for CTD data extraction.

| **Information** | **Regex Pattern** | **Example** |
| --- | --- | --- |
| Drug | (.*?)\sbinds\sto\s. | **Permethrin** results in increased expression of CHK-1 mRNA |
| Gene | (.*?)\s(protein\s\|gene\s\|mRNA\s\|.*\s) | **NRAS** gene mutant form results in increased susceptibility to Methotrexate |
| Relation | results\sin\s(.*?)(?:of\|to)\s(?:.*) | 10-decarbamoylmitomycin C results in **increased stability of** TP53 protein |

**CIViC - Data Extraction and Integration**

The CIViC database provides detailed information on genes, variations, diseases, drugs, and associated text snippets from scientific publications (nightly-ClinicalEvidenceSummaries.tsv). Processing the CIViC data involves two key steps: filtering and manual curation.

During the filtering step, we retained only records with non-empty values for all four key entities—gene, variation, disease, and drug—resulting in 2,586 relevant records.

Next, the curation step involved reviewing the text snippets extracted from scientific publications. We assessed whether these snippets contained meaningful information about drug sensitivity or mechanisms. Sentences meeting these criteria were retained for further analysis and extracted the relevant relationships.

**TTD - Data Extraction and Integration**

The Therapeutic Target Database (TTD) is a comprehensive resource that provides detailed information on drug targets. Data were downloaded from the TTD search page (<http://db.idrblab.net/ttd/search/ttd/drm-drug>) and then processed and manually reviewed for accuracy.

The dataset, including drug names, disease names, genetic variations, and species information, was organized into a structured format using a pandas DataFrame. We further refined the data by filtering out non-human records, retaining only entries related to Homo sapiens. This step ensured that the dataset was relevant for human drug sensitivity studies.

**DCDB - Data Extraction and Integration**

The DCDB dataset was first downloaded in its entirety from the official repository. As the data was spread across multiple tables, we merged these tables into a cohesive dataset by aligning records based on key identifiers such as drug IDs, combination IDs, and source references.

After merging, the records related to drug therapies were carefully organized and cleaned to ensure accuracy and usability.

**PharmacotherapyDB -Data Extraction and Integration**

The PharmacotherapyDB dataset was downloaded directly from its official GitHub repository (<https://github.com/dhimmel/indications>). This database offers detailed information on drug indications, which is crucial for understanding therapeutic practices.

After downloading, we merged data from various sources within the PharmacotherapyDB to consolidate information on drug indications from multiple studies and databases, creating a comprehensive dataset.

To ensure data quality, we performed a deduplication process, identifying and removing any duplicate records that may have resulted from the consolidation of multiple sources.

**DoCM - Data Extraction and Integration**

The variants_literature file from DoCM contains 183 records derived from 143 different publications. We manually curated these records, meticulously identifying and documenting the drugs, diseases, variations, and their interrelationships as reported in the literature.
